# Levels of matrix metalloproteinase-8 and cold test in reversible and irreversible pulpitis

**DOI:** 10.1097/MD.0000000000023782

**Published:** 2020-12-24

**Authors:** Eva Concepción Aguirre-López, Nuria Patiño-Marín, Gabriel Alejandro Martínez-Castañón, Carlo Eduardo Medina-Solís, Brenda Eréndida Castillo-Silva, Oscar Cepeda-Argüelles, Luis Alejandro Aguilera-Galaviz, Pedro Rosales-García

**Affiliations:** a Program of Doctorate in Dental Sciences, Department of Clinical Research; b Program of Doctorate in Dental Sciences, University of San Luis Potosí, San Luis Potosí, SLP; c Department of Dentistry, Institute of Health's Sciences, Autonomous University of The State of Hidalgo, Pachuca, Hidalgo; d Master in Stomatology, Benemerita Autonomous University of Puebla, Puebla; e Master in Biomedical Sciences, Autonomous University of Zacatecas, Zacatecas, México.

**Keywords:** cold test, matrix metalloproteinase-8, pulpitis

## Abstract

Researchers have reported false positive/negative results of the cold test in the diagnosis of pulpitis. Knowledge of the correlation between results of the cold test and proteins could aid in decreasing the frequency of incorrect diagnosis. To associate the levels of matrix metalloproteinase-8 (MMP-8) with the responses (in seconds) to the cold test in teeth diagnosed with reversible and irreversible pulpitis.

A cross-sectional study was performed. A total of 150 subjects were evaluated, of which 60 subjects met the selection criteria. The participants were divided into 3 groups: Group 1, healthy pulps, 20 subjects with 20 posterior teeth (premolars) with clinically normal pulp tissue; Group 2, reversible pulpitis, 20 patients with 20 teeth diagnosed with reversible pulpitis; and Group 3, irreversible pulpitis, 20 subjects with 20 teeth diagnosed with irreversible pulpitis. All participants were evaluated based on the following variables: medical and dental history, cold test, and expression of MMP-8 by enzyme-linked immunosorbent assay in dentin samples.

Responses to the cold test between 4 to 5 seconds (second evaluation; *P* < .0001) were associated with high levels of MMP-8 (mean, 0.36 ng/mL) in the reversible pulpitis group. In the irreversible pulpitis group, the responses from 6 to ≥10 seconds (second evaluation; *P* < .0001) were associated with a higher average of MMP-8 levels (mean, 1.97 ng/mL).

We determined that an increase in the duration of response to the cold test was associated with an increase in MMP-8 levels (Rho = 0.81, *P* < .0001) in teeth with pulpitis. The above correlations can be considered an adjunct to the clinical diagnosis of pulpitis.

## 1 Introduction

### 1.1 Cold test

Clinical diagnosis is a set of variables used by health professionals to determine the presence of a disease. The cold test is a pulp sensitivity test used for the diagnosis of pulpal conditions.^[1]^ This test is routinely performed in clinical practice because of its simplicity and low cost. Pulp sensitivity tests are based on subjective response of the patient triggered by an external stimulus (cold) on the nervous system. The presence of a vital or necrotic pulp is identified by the cold test.^[1–3]^ A vital pulp responds positively to the cold test due to the presence of vital nerve fibers, whereas a necrotic pulp does not show any response. Therefore, the cold test evaluates the response of nerves in the pulp, and not the pulp vascularity, which is an indicator of pulp vitality.^[1–3]^ Several researchers have reported false positive/negative results of pulp sensitivity tests, which could lead to incorrect diagnosis of pulpitis. The placement sites for the test, tooth characteristics, presence of diseases in the teeth, and treatments are associated with incorrect results.^[2–14]^

### 1.2 Reversible and irreversible pulpitis

Reversible and irreversible pulpitis are conditions associated with inflammation of the dental pulp tissue. Inflammatory processes that occur in the pulp tissue are complex and release an assortment of chemical mediators. Vasodilatation, increased vascular permeability, and leukocyte extravasation are vascular changes associated with the development of pulpal inflammation.^[1–3]^ Cold tests do not identify the presence or the degree of inflammation in the pulp. Dental history, clinical examination, radiographic analysis, and pulp sensitivity tests are used in the clinic to identify pulpal inflammation.^[1–3]^ Therefore, it is necessary to conduct clinical studies that provide information and identify pulpal inflammation to reduce the frequency of incorrect diagnosis. Molecular methods such as analysis of protein expression (such as matrix metalloproteinase-8 [MMP-8]) could be an option to reduce the frequency of false responses to the cold test in clinical practice.

### 1.3 Molecular methods: MMP-8

Different protease systems, such as aspartic proteinases, MMPs, and serine proteinases regulate the extracellular matrix (ECM) of tissues under both physiological and pathological conditions. MMP family proteins play dual roles in the pathogenesis, they stimulate protective innate and/or adaptive immune responses, as well as cause tissue destruction. The collagenases-1, -2, and -3 (MMP-1, MMP-8, and MMP-13, respectively) belong to the class of MMPs.

Dentin matrix formation, dental caries, and secondary dentin formation are associated with the presence of MMP-8 in the tooth. Some mechanisms of MMP-8 in degradation of the dentinal ECM and pulp tissue are as follows: release of enzymes by host and bacterial cells, phagocytosis of matrix components, release of reactive oxygen species, and release of cytokines and inflammatory mediators.^[15–22]^

Results of the correlation between pulp sensitivity tests and levels of MMP-8 could be considered in clinical practice as reference values, to reduce the frequency of false responses in the diagnosis of pulpal conditions. Therefore, the aim of this study was to associate the responses (in seconds) to the cold test with levels of MMP-8 (in dentin samples) in teeth diagnosed with reversible and irreversible pulpitis. The results could be used as an adjunct to the clinical diagnosis of a healthy pulp, and reversible or irreversible pulpitis, especially in challenging cases.

## 2 Methods

This cross-sectional study was performed from January 2018 to April 2019 in San Luis Potosi, Mexico. Based on the ethical principles of the Declaration of Helsinki, informed and voluntary written consent was obtained from the patients prior to the start of the study. The Research Ethics Committee of the Autonomous University of San Luis Potosi (code: CONBIOÉTICA24CE101320150526) approved this study.

### 2.1 Study population

**Figure 1:**
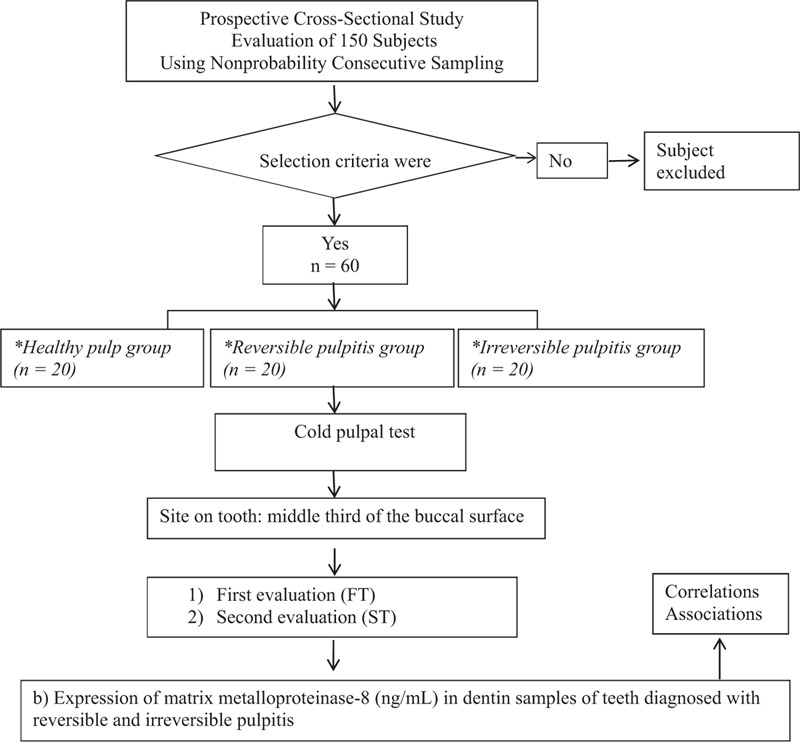
Study protocol. ^∗^Based the criteria published by the American Association of Endodontists and by opening the tooth cavity, we were able to confirm the diagnosis in the study groups; FT = seconds from application of the stimulus until the participant raised a hand. ng/mL = nanograms per millilitre. ST = seconds from removal of the stimulus until disappearance of the sensation.

A total of 150 subjects who visited the dental clinic at the Autonomous University of San Luis Potosi were evaluated to identify the subjects who met the selection criteria. All participants were selected using the nonprobability consecutive sampling technique (Fig. 1).

### 2.2 Calculating sample size

A comparison of means was used to calculate the sample size. The calculations were based on a previous study,^[18]^ considering the difference in MMP-8 levels (65% of difference) between diseased and healthy teeth. The smallest sample size obtained was 20 participants for each group with a power of 0.80 and a significance level of 0.05 (Fig. 1).

### 2.3 Selection criteria

A total of 60 subjects were included in this study. The inclusion criteria were as follows: subjects without any diagnosed systemic diseases, both sexes, age 18–65 years, no history of allergies, nonsmokers, no drug use (narcotics, alcohol, or nonsteroidal anti-inflammatory drugs) for 24 hours before the study, subjects with posterior teeth (premolars) with clinically normal pulp tissue indicated for orthodontic extraction, and subjects with a diagnosis of reversible or irreversible pulpitis. Exclusion criteria were as follows: pregnancy, teeth with full-surface crowns, restorations in the surfaces under evaluation, cavity inaccessibility, calcified root canals, and incomplete root formation.

### 2.4 Medical and dental history

An investigator recorded the medical history, including details of any medication taken within the past 6 months. The following variables pertaining to dental history were recorded: treatments undergone (past and recent), pain (onset, duration, frequency, intensity, and location), clinical evaluation (caries, restorations, enamel loss [abrasion, attrition, or fractures]), radiographic analysis (size and shape of the pulp chamber and root canals, radiolucent lesions, and thickening of periodontal ligament), and periodontal evaluation (periodontal probing, gingivitis, and abscesses).^[3]^

### 2.5 Study groups

Subjects who met the predetermined criteria were categorized into 3 groups. Group 1 represented healthy pulps and comprised 20 subjects with 20 posterior teeth (premolars) with clinically normal pulp tissue indicated for orthodontic extraction, with an apparently healthy crown (without enamel loss, restorations, or caries), without periodontal disease, asymptomatic; and without radiographic evidence of periapical pathology. Group 2 represented reversible pulpitis and comprised 20 patients with 20 teeth diagnosed with reversible pulpitis; patients with no clinical history of spontaneous pain; those with a normal reaction or hypersensitivity to percussion; and those with no radiographic evidence of periradicular pathosis. Group 3 represented irreversible pulpitis and comprised 20 patients with 20 teeth diagnosed with irreversible pulpitis; patients with moderate-to-severe spontaneous pain of approximately 24 hours duration; those with a normal reaction or hypersensitivity to percussion; and those with no radiographic evidence of periradicular pathosis.^[1,23,24]^ Diagnoses in the study groups were confirmed by opening the tooth cavity (Fig. 1). All participants were evaluated based on the 2 main dependent variables: responses to the cold pulpal test and MMP-8 levels in the dentin samples of teeth diagnosed with reversible and irreversible pulpitis (Fig. 1).

#### 2.5.1 Cold pulpal test

A clinical researcher isolated the teeth using cotton rolls and sprayed a No. 2 cotton pellet with a refrigerant spray (1, 1, 1, 2-tetrafluoroethane, Hygienic Endo-Ice Green [Endo-Ice]; Coltene Whaledent, Cuyahoga Falls, OH),^[3,4,6]^ which was then placed on the crown of the tooth (at the middle third of the buccal surface) for 25 seconds or until the subject raised a hand to indicate cold sensation. The results of the test were reported at 2 different times: first evaluation: seconds from application of the stimulus until the subject raised a hand and second evaluation: seconds from removal of the stimulus until disappearance of the sensation (Fig. 1).^[3]^

*Application of the test*. The test was performed by a single researcher. The researcher was blinded to the clinical signs and symptoms, dental history, periodontal status, and radiographic findings of the subjects. Participants were instructed to raise their hands the moment they felt a sensation during testing and to lower their hands the moment the sensation disappeared.^[3]^

#### 2.5.2 Expression of MMP-8 in dentin

*Obtaining dentin samples from the study groups*. Dental treatment was performed after completion of the test in the groups, following anesthesia (with 2% lidocaine and 1:100,000 diluted epinephrine) and isolation with a rubber dam.^[3,25]^

*Obtaining dentin samples from the reversible pulpitis group*. The enamel was removed and dental caries were eliminated with a diamond bur under continuous water spray. Opening of the cavity did not include the pulp chamber (confirming the diagnosis of reversible pulpitis), and the dentin sample was extracted using a dental excavator (Hu-Friedy).^[26–28]^

The samples were placed in a conical tube (Eppendorf) with physiological serum and stored at −20 °C until processing. We continued with the pulpal coating treatment after obtaining the sample.

*Obtaining dentin samples from the irreversible pulpitis group*. Similar to the reversible pulpitis group, the enamel and dental caries were eliminated with a diamond bur. Opening of the cavity included the pulp chamber (bleeding within the pulp chamber confirmed the diagnosis of irreversible pulpitis).^[2,3,27,29]^ The dentin sample was extracted using a dental excavator and stored following the previously explained procedure.^[26,28]^ The treatment was completed after obtaining the sample.

Obtaining dentin samples from the healthy pulp group (teeth indicated for orthodontic extraction). Extraction was carried out within 3 minutes.^[30,31]^ Immediately after extraction, the teeth were cleaned of blood with distilled water, and any attached soft tissue was carefully scraped with a scalpel to remove remnants of the periodontal ligament. The teeth were sectioned using a cylindrical diamond bur in a high-speed handpiece irrigated with saline solution.^[26,31]^ The dentin sample was extracted without the pulp chamber using a dental excavator. Samples were stored following the previously explained procedure. Once the extraction was completed, the patients continued and ended the treatment.^[31,32]^

#### 2.5.3 Expression of MMP-8 by enzyme-linked immunosorbent assay (ELISA)

##### 2.5.3.1 Preparation of mineralized dentin powder

Dentin samples were reduced to a fine powder by freezing the fragments in liquid nitrogen and triturating them using a steel mortar/pestle.^[20]^

##### 2.5.3.2 Demineralization procedure

Dentin powder was demineralized with 0.5 mol/L ethylenediaminetetraaceticacid in four cycles (96 hours per cycle) to extract the mineral-associated proteins. The extracts were then centrifuged and washed with distilled water. The dentin samples were kept frozen until use.^[26,33]^

##### 2.5.3.3 Extraction of proteins from the dentin samples

Protein extraction was performed using TRIzol reagent (Sigma–Aldrich Chemical Co., St. Louis, MO). The reagent is used for the extraction of RNA as well as DNA and proteins from tissues of human origin.^[34,35]^ Guanidine isothiocyanate is a component of TRIzol and an effective protein denaturant.^[26,33,35,36]^ Dentin dust was resuspended in 0.5 mL TRIzol, and the proteins were precipitated with isopropanol. The protein pellets were washed thrice with 0.3 M guanidine hydrochloride (Sigma–Aldrich) and 95% ethanol (Sigma–Aldrich), centrifuged, and washed with absolute ethanol (Sigma–Aldrich). The pellets were then dissolved in 50 μL urea-CHAPS (9.5 M urea/2% CHAPS; Sigma–Aldrich). Protein quantification was performed using the Bradford microplate assay. The protein solutions were aliquoted and stored at −20 °C before use.^[3,29,30]^

##### 2.5.3.4 Determination of MMP-8 levels by ELISA

ELISA, using a commercially available kit (MyBioSource, Inc., San Diego, CA), was performed to measure the concentration of MMP-8 in the dentin. The assay was performed according to the manufacturer's instructions. We used 10 ng of total protein extracted from the dentin of each sample to ensure homogeneity. Samples were assayed in groups of 3, and mean values were calculated in ng/mL. The intensity of the color determined by spectrophotometry was proportional to the amount of human MMP-8 present in each well during immunological incubation (Fig. 1).^[3,26]^

### 2.6 Statistical analyses

Categorical variables were reported as frequencies and percentages, while continuous variables were reported as means and standard deviations. Shapiro-Wilk and Brown-Forsythe tests were performed to determine the distribution of the variables. We used the Kruskal–Wallis test and Mann–Whitney *U* test to establish the differences between groups. Spearman rank correlation coefficient was used to identify correlations.^[37]^ Data were analyzed using JMP ver. 9.0 (SAS Institute, Cary, NC) and Stata ver. 11.0 (Stata Corp LP, College Station, TX) statistical software.

## 3 Results

**Figure 2:**
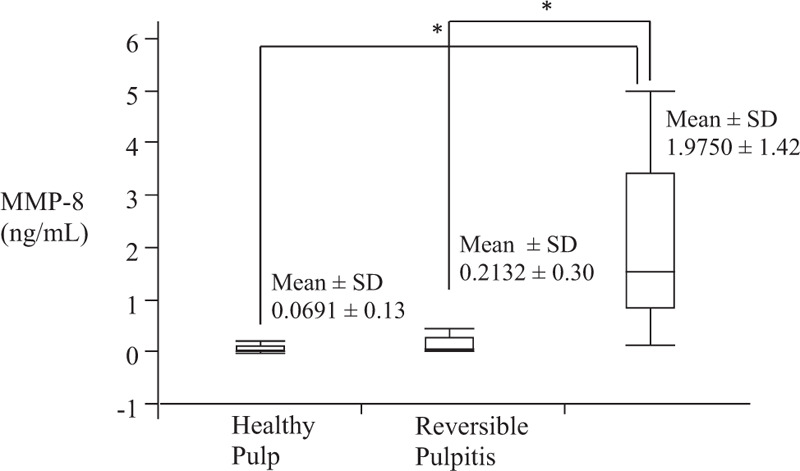
Levels of matrix metalloproteinase-8 (MMP-8) in the study groups. n = 60 teeth (20 teeth per group); ng/mL = nanograms per millilitre; SD = standard deviation; Kruskal-Wallis test and ^∗^Mann–Whitney *U* test: *P* = .0001.

Out of the 150 subjects evaluated, 60 met the selection criteria. The patients were divided into 3 groups: 20 patients (45% women) with 20 teeth diagnosed with healthy pulps, aged 18–50 years (mean = 28, standard deviation [SD] = 8.6), 20 patients (70% women) with 20 teeth with reversible pulpitis, aged 20 to 62 years (mean = 33, SD = 12.3), and 20 patients (70% women) with 20 teeth with irreversible pulpitis, aged 23 to 65 years (mean = 44, SD = 12.8). One tooth per patient was considered in the 3 groups. Table 1 shows the types of teeth and their clinical evaluation. Premolars (85% with healthy pulps) and maxillary molars (40% with reversible pulpitis and 30% with irreversible pulpitis) were the teeth with the highest frequencies in all groups. In the groups with pulpitis, dental caries (100%) was the predominant pathology. MMP-8 levels in the study groups are shown in Fig. 2. The lowest mean MMP-8 concentration was observed in the group with healthy pulps (0.0691 ng/mL) and the highest was in those with irreversible pulpitis (1.9750 ng/mL). We observed statistically significant differences (*P* = .0001) in the levels of MMP-8 between the irreversible pulpitis and the healthy pulp groups (Rho = 0.8277, *P* = .0001), and the reversible pulpitis group (Rho = 0.7646, *P* = .0001), with a higher average concentration of MMP-8 in the irreversible pulpitis group. Table 2 shows the levels of MMP-8 and responses to the cold test in teeth with healthy pulps. We identified the highest response frequencies, both at the first and second evaluations during the test, at 1 and 2 seconds, with MMP-8 levels between 0.0282 and 0.1140 ng/mL. The levels of MMP-8 and responses to the cold test in teeth with reversible and irreversible pulpitis are shown in Table 3. As in the healthy pulp group, during the first evaluation, we identified the highest response frequencies at 1 and 2 seconds in teeth with pulpitis (MMP-8 levels between 0.1000 and 0.3945 ng/mL). However, during the second evaluation, the highest response frequencies were identified at 3, 4, and 5 seconds in teeth with reversible pulpitis, with values of MMP-8 between 0.0265 and 0.4917 ng/mL. In relation to the irreversible pulpitis group, we observed response frequencies during the test at 6 to ≥10 seconds with values of MMP-8 between 0.8699 and 3.9829 ng/mL.

**Table 1:** Types of teeth and their clinical evaluation in the study groups.

Jaw	Types of teeth	Healthy pulp(n = 20)	Reversible pulpitis(n = 20)	Irreversible pulpitis(n = 20)
			Frequency (%)	
Maxilla	Anterior	–	3 (15)	5 (25)
	Premolar	17 (85)	2 (10)	1 (5)
	Molar	–	8 (40)	6 (30)
Mandible	Anterior	–	–	1 (5)
	Premolar	3 (15)	3 (15)	2 (10)
	Molar	–	4 (20)	5 (25)
Clinical evaluation	Healthy^∗^	20 (100)	–	–
	Caries	0	20 (100)	20 (100)
	Restoration	0	6 (30)	4 (20)
	Enamel loss	0	2 (10)	3 (15)

**Table 2:** Levels of MMP-8 and responses (in seconds) to the cold test in teeth with healthy pulps.

Cold testFirst evaluation(seconds)	Frequency of response of teeth (%)	Levels of MMP–8 (ng/mL)Mean
1	7 (35)	0.0775
2	10 (50)	0.0282
3	2 (10)	0.1179
4	1 (5)	0.3224
Second evaluation, s		
1	9 (45)	0.0425
2	8 (40)	0.1140
3	3 (15)	0.0293

**Table 3:** Levels of MMP-8 and responses (in seconds) to the cold test in teeth with reversible and irreversible pulpitis.

	Reversible pulpitisn = 20		Irreversible pulpitisn = 20	
Cold test	Frequency of response of teeth (%)	Levels of MMP-8 (ng/mL) mean	Frequency of response of teeth (%)	Levels of MMP-8 (ng/mL) mean
First evaluation, s				
1	11 (55)	0.1000	10 (50)	2.7672
2	8 (40)	0.3945	6 (30)	1.1124
3	1 (5)	0.0088	2 (10)	1.6891
4	–	–	2 (10)	0.8882
Second evaluation, s				
1	–	–	–	–
2	1 (5)	0.0088	–	–
3	7 (35)	0.0265	–	–
4	7 (35)	0.2303	–	–
5	5 (25)	0.4917	–	–
6–7	–	–	9 (45)	0.8699
8–9	–	–	6 (30)	1.9595
≥10	–	–	5 (25)	3.9829

### 3.1 Analysis of the 3 study groups during the first evaluation

We did not observe any statistically significant differences (*P* > .05) between the seconds in the groups on comparing the averages (mean = 1.85 in the group with healthy pulps, 1.50 in reversible pulpitis, and 1.80 in the irreversible pulpitis group) of the different responses (from 1 to 4 seconds) and the study groups during the first evaluation. Moreover, we did not find a correlation (Rho = 0.0200, *P* = .8466) between the responses in seconds and the values of MMP-8 in the 3 groups.

### 3.2 Analysis of the 3 study groups during the second evaluation

On analysis of the same variables during the second evaluation, we observed an association (*P* < .0001) between the average (mean = 1.70 in the group with healthy pulps, 3.80 in reversible pulpitis, and 9.40 in the irreversible pulpitis group) of the different responses (from 1 to ≥10 seconds) and the study groups (n = 60). Values from 6 to ≥10 seconds (mean = 9.40) were associated with the irreversible pulpitis groups. Furthermore, we observed a *P* < .0001 and Rho = 0.81 on correlating seconds of responses with levels of MMP-8 in the 3 study groups. Therefore, the increase in seconds was associated with an increase in MMP-8 levels in the teeth with pulpitis. We compared the values of MMP-8 with the groups with responses from 1 to 3 seconds (n = 28, 20 teeth diagnosed with healthy pulps and 8 with reversible pulpitis) and the group with responses from 4 to 5 seconds (12 teeth diagnosed with reversible pulpitis). We identified a correlation (Rho = 0.70, *P* < .0001) and an association (*P* < .0001), and high values of MMP-8 (mean = 0.3607 ng/mL) in the reversible pulpitis group with responses from 4 to 5 seconds. We also compared the values of MMP-8 with the groups with responses from 1 to 5 seconds (n = 40, 20 teeth diagnosed with healthy pulps and 20 with reversible pulpitis), and the group with responses from 6 to ≥10 seconds (20 teeth diagnosed with irreversible pulpitis). We observed a correlation (Rho = 0.80, *P* *<* .0001) and an association (*P* *<* .0001), and high values of MMP-8 (average 1.9750 ng/mL) in the irreversible pulpitis group with responses from 6 to ≥10 seconds.

## 4 Discussion

The aim of this study was to associate the responses to the cold test with levels of MMP-8 in teeth diagnosed with reversible and irreversible pulpitis. This analysis would enable identification of a possible application in clinical practice.

### 4.1 Cold test

Diagnosis consists of gathering information to identify the absence or presence of an illness. Medical and dental history, clinical examination, pulp sensitivity tests, and radiographic analysis are necessary for the diagnosis of pulpal conditions.^[1]^ Cold test is a pulp sensitivity test routinely performed in clinical practice due to its simplicity and low cost. The results of the test are based on subjective response of the patient triggered by an external stimulus (cold) on the nervous system. A clinically healthy pulp is asymptomatic, producing a mild-to-moderate transient response to cold stimulus. The response subsides within a few seconds after removal of the stimulus. In reversible pulpitis (localized inflammation), the stimulus causes a sharp pain that subsides on removal of the stimulus or within a few seconds; however, the acutely inflamed pulp is associated with severe symptoms in irreversible pulpitis (advanced inflammation). Temperature changes elicit a sharp pain followed by a dull prolonged ache.^[1]^ In clinical practice, pulp sensitivity tests do not identify the presence or the degree of pulpal inflammation.^[1–3]^ However, despite their limitations (false responses), thermal tests are useful in the diagnosis of pulpal conditions considering the differences in test response of diseased and healthy pulps.^[1]^ Sex, age, placement site for the test, tooth type, multirooted teeth, presence of caries, smaller pulp chambers, calcifications, periodontal pocket and attachment loss, enamel loss, necrotic teeth, extensive restorations, and asymptomatic teeth are variables associated with false responses to thermal tests.^[2–14,38]^ Therefore, it is necessary to propose strategies (reference values) or tools (molecular methods) that reduce the frequency of false responses.

### 4.2 Levels of MMP-8 in dentin

#### 4.2.1 MMP-8

Different protease systems, such as aspartic proteinases, serine proteinases, and MMPs regulate the ECM of tissues in both physiological and pathological conditions.

MMPs are a group of >25 secreted enzymes produced by connective tissue cells (fibroblasts, osteoblasts, and odontoblasts), as well as by polymorphonuclear leukocytes and other inflammatory cells. MMPs participate in normal tissue modeling. They are also involved in pathological conditions such as inflammation and degradation of the bone, autoimmune diseases, and invasion and migration of cancer cells across the basement membrane (tumor metastasis).

MMPs are classified into 5 main classes: collagenases, gelatinases, stromelysins, matrilysins, and membrane-type MMPs. Their role in tissue destruction is evident; however, it is not completely clear. The expression of MMPs is regulated by proinflammatory cytokines and growth factors as well as ECM components. Collagenases include MMP-1, MMP-8, and MMP-13. Collagenases and gelatinases, which tend to break down collagens and laminins, are considered the key ECM MMPs.

#### 4.2.2 MMP-8 in dentin ECM

MMPs (MMP-8, -2, -9, -3, -2, -14, and -20) have been isolated from the dentin, pulp tissue, and odontoblasts, where they play an important role in formation of the dentinal matrix, modulation of caries progression, and secondary dentin formation.

##### 4.2.2.1 Dentin matrix

The dentinal ECM consists mainly of type I (90%), as well as type III and V collagen fibrils (1%–3%), which undergo fibrillogenesis to form a template that can be effectively mineralized. ECM can be degraded by various mechanisms that include release of enzymes by host and bacterial cells, phagocytosis of matrix components, release of reactive oxygen species, and release of cytokines, inflammatory mediators, and apoptotic proteins that influence enzymes connected with different components. In relation to the presence of caries, the destruction of dentin can be divided into 2 distinct steps: acid dissolution of the mineralized portion of the tooth and degradation of the dentinal collagen matrix by action of proteolytic enzymes. Some mechanisms by which MMP-8 contributes to collagen degradation in the dentin are as follows: dissolution of hydroxyapatite by acidic action of bacteria as well as other ECM components, salivary pH of 4 or 5, repeated increase in the pH due to cyclical pH fluctuation in caries, and further degradation of the fragmented collagen-gelatin by gelatinases (MMP-2 and -9) or nonspecific proteases of bacterial or endogenous origin.^[15–22]^

### 4.3 Association between the cold test and MMP-8 levels in teeth diagnosed with reversible and irreversible pulpitis

The results of the test were reported at 2 different times (first and second evaluations), and the concentration of MMP-8 was evaluated from carious dentin samples of teeth diagnosed with reversible and irreversible pulpitis (MMP-8 was not identified in the pulp because it was not feasible to obtain pulp samples from the reversible pulpitis group).

The following observations were reported after analysis of the variables in the study: the increase in seconds (second evaluation) was associated with an increase in the MMP-8 levels (Rho = 0.81, *P* < .0001) in teeth with pulpitis, high values (*P* < .0001) of MMP-8 (mean, 0.3607 ng/mL) were observed during the second evaluation in the reversible pulpitis group (from 4 to 5 seconds), and high values of MMP-8 (average, 1.9750 ng/mL) (Rho = 0.80, *P* < .0001) were observed in the irreversible pulpitis group (from 6 to ≥10 seconds).

We performed a scientific text search with the variables cold test and MMP-8 to compare the results of this study with those previously reported in the literature. However, we were unable to find any information related to this topic. On the contrary, in a different study, we identified the relationship between the expression of calcitonin gene-related peptide (CGRP) and the responses to pulp sensitivity tests in subjects with healthy pulps and irreversible pulpitis. The following were observed on comparison of the groups: the increase in seconds was associated with an increase in the expression of CGRP and responses to the test of ≥4 seconds correlated with the presence of illness (irreversible pulpitis) during the second evaluation.^[3]^ The above results are consistent with those obtained in this study.

### 4.4 Clinical application of the association between the cold test and MMP-8 levels in teeth diagnosed with reversible and irreversible pulpitis

False responses to the cold test have been reported by several researchers.^[2,3,5,8,9,10,12,13]^ Therefore, it is important to identify strategies (reference values) or tools (molecular methods) to complement or confirm the diagnosis of pulpal conditions. In this study, test responses were associated and correlated with the collagenase, MMP-8, which is related to tissue inflammation and destruction. The results obtained could be considered in routine clinical practice as reference values, and can be used to initiate studies on diagnostic tests.

#### 4.4.1 Clinical applications of the cold test

Associations and correlations were identified during the second evaluation (seconds from removal of the stimulus until disappearance of the sensation) in the cold test. Thus, the clinical applications of the test are as follows: patient response at ≥4 seconds during the test could suggest a possibility of the presence of an illness (pulpitis), patient response between 4 and 5 seconds could suggest a possibility of reversible pulpitis, and patient response between 6 and ≥10 seconds could be associated with a diagnosis of irreversible pulpitis.

The aforementioned correlations and associations can be used to complement the diagnosis of a healthy pulp, and reversible or irreversible pulpitis, especially in cases that are difficult to diagnose. However, further studies on diagnostic tests are needed to confirm the results. The use of biomarkers with molecular techniques could identify the state of inflammation of the pulp tissue. The correlation of the different biomarkers with pulp sensitivity test is a strategy to confirm the results of the present study.

The following can, therefore, be concluded: we did not observe responses of ≥4 seconds during the second evaluation in the group with healthy pulps. The increase in seconds was associated with an increase in MMP-8 levels (Rho = 0.81, *P* < .0001) in teeth with pulpitis. We observed high values of MMP-8 in the reversible pulpitis group from 4 to 5 seconds (*P* < .0001). High values of MMP-8 were observed in the irreversible pulpitis group from 6 to ≥10 seconds (*P* < .0001). Therefore, a possibility of pulpitis could be considered with a test response ≥4 seconds. Furthermore, there could be a possibility of reversible pulpitis with a response time between 4 and 5 seconds, and that of irreversible pulpitis with a response time between 6 and ≥10 seconds.

## Acknowledgments

The Ministry of Education, Mexican Federal Government, through the Faculty Development Program (PRODEP) supported this publication.

## Author contributions

**Conceptualization:** Eva Concepción Aguirre-López, Nuria Patiño-Marín, Carlo Eduardo Medina-Solís, Brenda Eréndida Castillo-Silva.

**Data curation:** Eva Concepción Aguirre-López, Nuria Patiño-Marín, Gabriel Alejandro Martínez-Castañón, Oscar Cepeda-Argüelles, Luis Alejandro Aguilera-Galaviz, Pedro Rosales-García.

**Formal analysis:** Nuria Patiño-Marín, Carlo Eduardo Medina-Solís.

**Funding acquisition:** Nuria Patiño-Marín, Gabriel Alejandro Martínez-Castañón, Brenda Eréndida Castillo-Silva, Oscar Cepeda-Argüelles, Luis Alejandro Aguilera-Galaviz.

**Investigation:** Eva Concepción Aguirre-López, Nuria Patiño-Marín, Gabriel Alejandro Martínez-Castañón, Carlo Eduardo Medina-Solís, Brenda Eréndida Castillo-Silva, Oscar Cepeda-Argüelles, Luis Alejandro Aguilera-Galaviz, Pedro Rosales-García.

**Methodology:** Eva Concepción Aguirre-López, Nuria Patiño-Marín, Gabriel Alejandro Martínez-Castañón, Carlo Eduardo Medina-Solís, Brenda Eréndida Castillo-Silva, Oscar Cepeda-Argüelles, Pedro Rosales-García.

**Project administration:** Eva Concepción Aguirre-López, Nuria Patiño-Marín, Carlo Eduardo Medina-Solís.

**Resources:** Brenda Eréndida Castillo-Silva, Luis Alejandro Aguilera-Galaviz.

**Software:** Gabriel Alejandro Martínez-Castañón, Carlo Eduardo Medina-Solís, Oscar Cepeda-Argüelles.

**Supervision:** Nuria Patiño-Marín, Gabriel Alejandro Martínez-Castañón, Carlo Eduardo Medina-Solís, Oscar Cepeda-Argüelles.

**Validation:** Nuria Patiño-Marín, Gabriel Alejandro Martínez-Castañón, Carlo Eduardo Medina-Solís, Brenda Eréndida Castillo-Silva, Oscar Cepeda-Argüelles, Luis Alejandro Aguilera-Galaviz, Pedro Rosales-García.

**Visualization:** Eva Concepción Aguirre-López, Gabriel Alejandro Martínez-Castañón, Carlo Eduardo Medina-Solís, Brenda Eréndida Castillo-Silva, Oscar Cepeda-Argüelles, Luis Alejandro Aguilera-Galaviz, Pedro Rosales-García.

**Writing – original draft:** Eva Concepción Aguirre-López, Nuria Patiño-Marín, Gabriel Alejandro Martínez-Castañón, Carlo Eduardo Medina-Solís, Brenda Eréndida Castillo-Silva, Oscar Cepeda-Argüelles, Luis Alejandro Aguilera-Galaviz, Pedro Rosales-García.

**Writing – review & editing:** Eva Concepción Aguirre-López, Nuria Patiño-Marín, Gabriel Alejandro Martínez-Castañón, Carlo Eduardo Medina-Solís, Brenda Eréndida Castillo-Silva, Oscar Cepeda-Argüelles, Luis Alejandro Aguilera-Galaviz, Pedro Rosales-García.
